# Sorption and Desorption of Vapor of n-Pentane by Porphyrin Aluminum Metal–Organic Framework: Mechanism of Bonding, Kinetics and Stoichiometry by Complementary *In-Situ* Time-Dependent and *Ex-Situ* Methods

**DOI:** 10.3390/nano13091529

**Published:** 2023-05-01

**Authors:** Georgia-Annicette Banga-Bothy, Alexander Samokhvalov

**Affiliations:** Department of Chemistry, Morgan State University, 1700 East Cold Spring Lane, Baltimore, MD 21251, USA

**Keywords:** metal–organic framework, sorption, controlled atmosphere, ATR-FTIR, *in-situ*, time-dependent, kinetics

## Abstract

Metal–organic frameworks (MOFs) are highly nanostructured coordination polymers that contain metal cations and organic linkers and feature very large pore volumes and surface areas. The sorption and desorption of n-pentane vapor by porphyrin aluminum metal–organic framework Al-MOF-TCPPH_2_ where TCPPH_2_ is tetrakis(4-carboxyphenyl)porphyrin linker were studied by a novel method of *in-situ* time-dependent attenuated total reflectance Fourier transform infrared (ATR-FTIR) spectroscopy in a controlled atmosphere and complementary *in-situ* and *ex-situ* methods. Sorption facilely occurs in the flow of dried air, and in the obtained adsorption complex the adsorbate molecules interact with phenyl and carboxylate groups of the linker and the O-H group. Sorption kinetics follows the pseudo-first-order rate law, as confirmed by *in-situ* time-dependent gravimetry. Further, an *ex-situ* (static) sorption of n-pentane vapor results in an adsorption complex with as much as 29.1 wt.% n-pentane with the stoichiometric formula [Al-MOF-TCPPH_2_]_2_(n-C_5_H_12_)_7_ and a distinct XRD pattern. Finally, in the flow of dried air, the adsorption complex gradually desorbed n-pentane, following the pseudo-first-order rate law. The reversibility of sorption and desorption makes porphyrin aluminum MOF promising for the separation of light hydrocarbons and chemo-sensing. *In-situ* time-dependent ATR-FTIR spectroscopy in a controlled atmosphere, in combination with *in-situ* time-dependent gravimetry, is a new approach for the determination of binding sites of sorbents with adsorbate molecules, the stoichiometry of complexes, and chemical kinetics of “solid–gas” interactions.

## 1. Introduction

Metal–organic frameworks (MOFs) are highly nanostructured coordination polymers that contain metal cations as “nodes” and organic linkers, and feature unusually large surface areas and pore volumes. This structure results in the capability of MOFs to sorb various molecules at high capacity and/or selectivity. In recent decades, MOFs have become a “hot” topic, and significant interest has been found in studies of heterogeneous catalysis, chemo-sensing, agriculture, purification of food, and biomedicine. As pertinent to sorption [1], areas of high interest in MOFs include gas separation [2] and storage [3], the sorption of toxic gases [4], sorption of water [5] e.g., the production of pure water from seawater [6] and from humid air [7].

Aluminum MOFs (Al-MOFs) form the group of MOFs, which attract an extensive interest for sorption in solutions [8] and the gaseous [9] phase. Al-MOFs are known for their good stability at high temperatures [10] and aggressive compounds in the form of liquid [11] and vapor [12]. The high stability of many Al-MOFs stems from the structure, which includes chemically very stable Al^3+^ cations (which cannot be oxidized or reduced) and anions of carboxylic acids which are also stable, especially when they contain aromatic or hetero-aromatic rings.

Porphyrins are nitrogen heterocyclic compounds with large rings and high versatility of structure and properties. Namely, porphyrins contain polar hetero-aromatic rings, which favor the sorption of polar molecules, and their aromatic units are suitable for the sorption of non-polar hydrophobic compounds. Indeed, porphyrins have been widely investigated for the chemo-sensing of various molecules and ions [13]. Notably, structural derivatives of tetrakis(4-carboxyphenyl)porphyrin denoted TCPPH_2_ are quite stable in aggressive environments, such as those created by redox stress in photocatalytic water splitting [14].

Aliphatic hydrocarbons find extensive and diverse uses, especially in petroleum and the natural gas industry, and significant research interest. In addition, common industrial chemicals, including petrochemicals, can be used in terrorist attacks [15] or in military warfare. The sorption of hydrocarbons was studied on MOFs [16]. To our knowledge, there are no studies of the sorption of hydrocarbon vapor by porphyrin MOFs or porphyrin Al-MOFs.

Molecular spectroscopy is a major approach when learning about the mechanism of sorption [17] and, specifically, the interaction of certain functional groups in the sorbent with adsorbate molecules [18]. Infrared (IR) spectroscopy is well suited for the detection of functional groups in sorbents and adsorbate molecules [19], and it allows *in-situ* studies of “host–guest” interactions [20]. In the attenuated total reflectance Fourier transform infrared (ATR-FTIR) spectroscopy, the evanescent field of IR radiation comes in direct contact with solid or liquid specimen. When a specimen on ATR crystal is exposed to compound(s) of interest and its spectra are recorded, this is an *in-situ* ATR-FTIR spectroscopy that can find use in the research of sorption, e.g., [21]. Surprisingly, only very few papers report using this method to study the mechanisms of reactions of powders [22]. Recently, we reported an *in-situ* time-dependent ATR-FTIR spectroscopic study [9] to determine the molecular mechanism of sorption of water vapor in ambient air on a highly hygroscopic Al-MOF MIL-160(Al).

Using a controlled atmosphere allows to eliminate the effects of dust and ambient chemicals such as water vapor, oxygen, carbon dioxide, etc., as well as to protect an operator from toxic gases. Indeed, studies of reactions in a controlled atmosphere found use in catalysis [23], manufacturing electronic devices [24], solid-state chemistry and physics [25], environmental research [26], life sciences [27], the food industry [28] and work with hazardous materials [29]. There are no studies, to our knowledge, on the sorption of hydrocarbons using *in-situ* ATR-FTIR spectroscopy in a controlled atmosphere. The combination of spectroscopic and kinetic studies is very useful in the mechanistic research of heterogeneous catalysis [30]; it would also be of interest in mechanistic *in-situ* studies of sorption and desorption.

**Figure 1:**
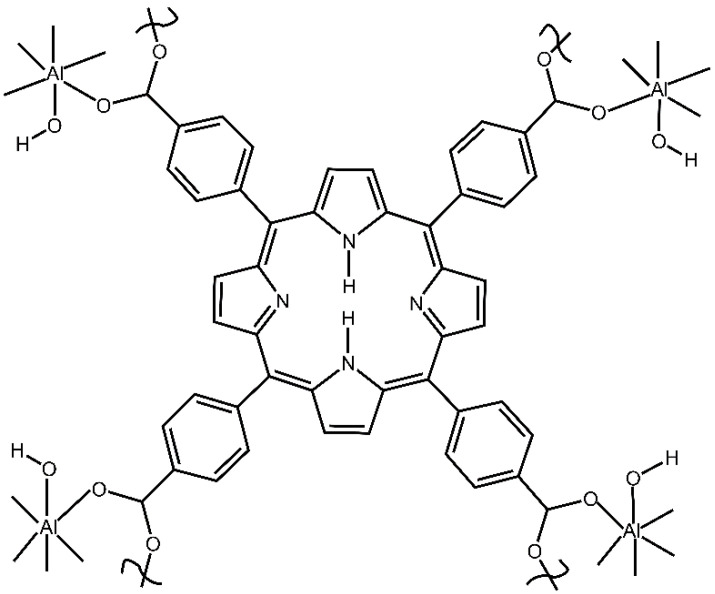
The simplified structural unit of compound **2** porphyrin aluminum metal–organic framework Al-MOF-TCPPH_2_ with Al^3+^ cations and TCPPH_2_ linker.

First, we studied the dynamic sorption of n-pentane vapor (in the flow of dried air) by mesoporous aluminum MOF, Al-MOF-TCPPH_2_, denoted here as compound **2** (Figure 1).

This was accomplished using a facile flow chamber for gas (vapor), which was conveniently added to the FTIR spectrometer with an ATR accessory, hence creating a controlled atmosphere for both *in-situ* and time-dependent experiments. Using this methodology, the binding sites of n-pentane adsorbate at specific functional groups in sorbent compound **2** were determined. Second, the kinetics of sorption was determined by *in-situ* time-dependent ATR-FTIR spectroscopy in a controlled atmosphere. Third, a complementary kinetic study was conducted by *in-situ* time-dependent gravimetry in a controlled atmosphere. Fourth, the sorption of n-pentane by compound **2** was also studied under *ex-situ* (static) conditions in saturated vapor. The obtained adsorption complex compound **3** was characterized by spectroscopic and structural methods, and its stoichiometry was determined. Finally, the kinetic study of the desorption of n-pentane from compound **3** was conducted by *in-situ* time-dependent gravimetry in a controlled atmosphere.

## 2. Materials and methods

### 2.1. Chemicals

The precursor compound of Al-MOF-TCPPH_2_ was tetrakis(4-carboxyphenyl)porphyrin (TCPPH_2_) of purity ≥ 97.0% from TCI Chemicals (Portland, OR, USA). Aluminum chloride AlCl_3_·6H_2_O of 99% purity was from Alfa Aesar (Tewksbury, MA, USA). Acetone (of reagent purity) was from Electron Microscopy Sciences (Hatfield, PA, USA). N,N-dimethylformamide (DMF) of ≥99.5% purity and n-pentane of >97% purity were from TCI Chemicals.

### 2.2. Synthesis of asisAl-MOF-TCPPH_2_ and Its Thermal Activation to actAl-MOF-TCPPH_2_

First, the as-prepared MOF asisAl-MOF-TCPPH_2_ was synthesized by the autoclave method, purified by washing with DMF and acetone [31], and the obtained sample (denoted for convenience compound **1**) was stored in a desiccator with molecular sieves. Second, the thermal activation of “starter” compound **1** was conducted [31]; notably, the freshly activated sample was quickly transferred to a glass jar (when still in the vented vacuum oven), then closed with a cap, and sealed with Parafilm tape. This resulted in a target compound, namely activated MOF actAl-MOF-TCPPH_2_ denoted here as compound **2**. The XRD patterns of both materials, asisAl-MOF-TCPPH_2_ and actAl-MOF-TCPPH_2_, were reported by us earlier [31].

### 2.3. Instrumental Characterization of Specimens

FTIR spectra were taken using the Nicolet IS10 spectrometer (Thermo Fisher Scientific, Waltham, MA, USA) which was operated in the ATR-FTIR mode. Figure S1 shows generic schematics (not to scale) of the ATR accessory.

The spectrometer was equipped with an ATR accessory model Golden Gate (from Specac Inc., Fort Washington, PA, USA, part number GS10500), which has diamond crystal. The data acquisition software of the FTIR spectrometer was OMNIC, in which a spectral resolution was set at 4 cm^−1^ and the optical aperture was at the “Open” parameter. To enable the *in-situ* time-dependent study of sorption, each spectrum was averaged 96 times (95.3 s or 1.5 min). To avoid the adverse effect of water vapor in ambient air on IR spectra, the FTIR spectrometer was continuously purged with an IR purge gas (dried air) at the rate of 30 scfh (standard cubic feet per hour) measured by a flow meter (model RMA-7 from Dwyer Instruments, Michigan City, IN, USA).

Dried air was produced by an FT-IR Purge Gas Generator (model 74-5041 Parker Balston, from Parker Hannifin Corporation, Haverhill, MA, USA) which contained a built-in air compressor. This setup allowed for the creation of dried air of spectroscopic quality with relative humidity RH < 1% and also free of carbon dioxide. To monitor the quality of FTIR spectra and further remove any remaining artifacts due to traces of water vapor, OMNIC data acquisition software had its parameter “Atmospheric Correction” enabled. The parameter “Spectral Quality Results” was set at “H_2_O level” ≥ 95%. The ATR-FTIR spectra were plotted in the absorbance mode. The numeric peak fitting of ATR-FTIR spectra was performed using the Microcal Origin 2016 program ( Origin Pro 2016 64-bit, from OriginLab, Northampton, MA, USA).

Powder X-ray diffraction (XRD) patterns were collected by a diffractometer model MiniFlex (from Rigaku Americas Corporation, The Woodlands, TX, USA) using a Cu K-alpha line at 0.15418 nm with increments of 2θ at 0.02 degrees.

### 2.4. Gas Flow Chamber Attachment on FTIR Spectrometer

Herein, the gas flow chamber attachment is denoted as the “flow chamber” for simplicity and is shown in Figure S2. The body of the flow chamber has walls (the left side, the right side, and the rear side), while its front side is the door for inserting the specimen. The flow chamber has a “roof” but no “bottom”, and its open bottom side is attached to the top panel of the FTIR spectrometer Nicolet IS10 and over the ATR accessory with a specimen. The process of fabrication of the flow chamber was described recently by us [32]. Outside of the flow chamber, the adapter had quick-disconnect termination for the connection of the gas inlet supply line.

Inside the flow chamber, a temperature and humidity sensor (data logger) was placed (model RC-4HC from Elitech Technology Inc., San Jose, CA, USA). The sensor was able to measure temperatures between −30 and +60 °C and relative humidity (RH) between 0 and 100% with ±5% accuracy in its low range. The USB cable from the sensor was withdrawn from the flow chamber via a wall cutout, and the sensor was logging the RH and temperature every few seconds to data logging software from Elitech Technology Inc. The drawing in Figure S2 was made using SketchUp Pro 2022 program (version 22.0.354 64-bit, from Trimble Inc., Westminster, CO, USA).

### 2.5. Setup for Preparation of Flow of Dried Air and Dried Air Saturated with n-Pentane

The flow of dried air (RH < 1%) was prepared by using an FT-IR Purge Gas Generator (model 74-5041 Parker Balston), and the stream of flow rate 5 scfh was withdrawn. A simple in-flow vapor saturation setup was essentially a gas flush can, namely a 250 mL Büchner flask which had a stopper with a glass tube protruding to the bottom. This gas flush was about ½ filled with liquid n-pentane, and dried air at a flow rate of 5 scfh was bubbled through liquid n-pentane.

### 2.6. Dynamic Sorption of n-Pentane Vapor by Compound ***2*** using In-Situ Time-Dependent ATR-FTIR Spectroscopy in Controlled Dry Atmosphere

A sample of compound **2** was placed on the ATR crystal, with a flow chamber attached to the FTIR spectrometer, and the door was closed. First, the chamber was pre-purged with dried air (RH < 1%) at a flow rate of 50 scfh, generated by an FT-IR purge gas generator (model 75-52 Parker Balston, from Parker Hannifin Corporation, Haverhill, MA, USA), equipped with an air compressor (model 8010A from California Air Tools, San Diego, CA, USA).

Then, the flow of dried air was reduced to 5 scfh, prepared by FT-IR Purge Gas Generator (model 74-5041 Parker Balston), and ATR-FTIR spectra were collected with settings as in Section 2.3. Next, the flow of gas through the chamber was switched to purge gas (dried air saturated with n-pentane vapor) at a flow rate of 5 scfh, see Section 2.5 and ATR-FTIR spectra were continuously collected with the same settings.

### 2.7. Dynamic Sorption of n-Pentane Vapor by Compound ***2*** Studied by In-Situ Time-Dependent Gravimetry

The setup in this work used an analytic balance, which was an improved modification of that reported by Padial et al. [33]. Namely, in Scheme S1 in ref. [33], the specimen was placed on a plate of analytical balance, and the flow of carrier gas with the vapor of a compound of interest was directed to the specimen, with continuous weight recording. However, the setup by Padial et al. [33] had the specimen open to ambient air, which may have resulted in the competitive sorption of the vapor of water from ambient air.

In this work, an analytical balance (model ME204T0, Mettler-Toledo, Columbus, OH, USA) with internal calibration and a USB data transfer interface to a PC was placed to the fume hood. First, plastic tubing with a ¼ inch OD from the gas flush was inserted inside an enclosure of the balance, with its end close to the plate with the specimen. Second, the top and side doors of the balance were closed, hence isolating its interior from the ambient atmosphere. The specimen of compound **2** was promptly spread on the tared watch glass (80 mm in diameter), then placed on the plate of analytical balance, and the doors of the balance were closed.

Then, the stream of dried air saturated with n-pentane vapor was passed over the specimen on the plate of balance. The setup allowed an *in-situ* gravimetric experiment to be conducted in a controlled atmosphere in the vicinity of the specimen. The experiment was conducted at a constant room temperature of 25 °C. The mass of the sample on the plate of balance was automatically transmitted every 3 sec from the balance to a lab PC via the USB port. In a reference experiment, a constant weight load on the plate of balance produced the same readings (within 0.001 g) over a few days.

### 2.8. Static (in Saturated Vapor) Sorption of n-Pentane by Compound ***2***

The sorption of n-pentane vapor was conducted in the vapor saturation chamber (closed desiccator) at room temperature. The setup was similar to that described by us [31] for water vapor sorption by this MOF. Major modifications were as follows: (a) instead of liquid water, liquid n-pentane was used, (b) the chamber did not contain a hygrometer/thermometer, (c) prior to inserting the specimen, the interior of this desiccator was purged with dried air, and (d) immediately after that, compound **2** on a quartz XRD sample plate was inserted into the chamber. The sorption chamber was closed overnight; the obtained sorption complex [Al-MOF-TCPPH_2_]_x_[n-C_5_H_12_]_y_ was denoted as compound **3**. Compound **3** on a quartz plate was removed from the chamber, promptly weighed, and returned to the chamber for storage. For instrumental analysis, compound **3** on a quartz plate was transferred to the XRD instrument or a small amount of it was placed on the ATR crystal of the FTIR spectrometer.

### 2.9. Dynamic Desorption of n-Pentane Vapor by Compound ***3*** Studied by In-Situ Time-Dependent Gravimetry

Most of the details are similar to those in Section 2.7, and the differences are as follows. Compound **3** (adsorption complex) on an XRD plate was placed on the plate of an analytical balance, the interior of the balance in contact with the specimen was purged with dried air at the flow rate of 5 scfh, and the mass of the specimen was automatically transmitted every 10 s to a lab PC.

## 3. Results and Discussion

### 3.1. Setting up the Flow Chamber for *In-Situ* Time-Dependent ATR-FTIR Spectroscopic Study of Sorption of n-Pentane Vapor by Compound ***2*** at Controlled Low Humidity

Before the start of the vapor sorption experiment, a sample of compound **2** was placed on ATR crystal; then the flow chamber was attached to the FTIR spectrometer, the door closed, and the flow chamber was pre-purged with dried air, see Materials and methods. During this pre-purge step, the relative humidity (RH) inside the flow chamber was recorded by the sensor every 10 s. First, the RH quickly decreased, Figure S3, then it remained low and constant at <5% (low end of sensor’s dynamic range, indicating low humidity of ca. 1%).

**Figure 2:**
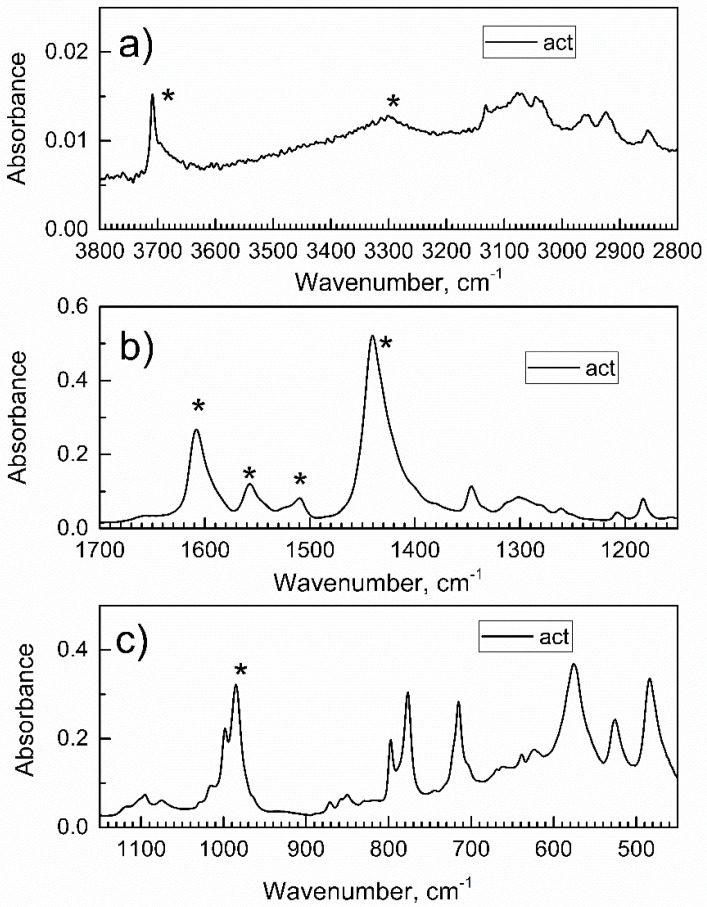
The survey *in-situ* ATR-FTIR spectra of the activated Al-MOF-TCPPH_2_ (compound **2**) in the flow of dried air. (**a**) The high wavenumbers IR range; (**b**) the mid-IR range; (**c**) the low wavenumbers IR range.

Simultaneously, the *in-situ* ATR-FTIR spectra of the specimen on the ATR crystal were recorded, Figure 2; they did not change during pre-purge with dried air (data not shown); hence, compound **2** was maintained in the activated (dry) state.

There were no peaks in the range 2800–1700 cm^−1^; peaks labeled with asterisks were the most characteristic ones, and they are assigned in [31].

### 3.2. Dynamics of Sorption of n-Pentane Vapor by Compound ***2*** Studied by In-Situ Time-Dependent ATR-FTIR Spectroscopy in Controlled Dry Atmosphere

**Figure 3:**
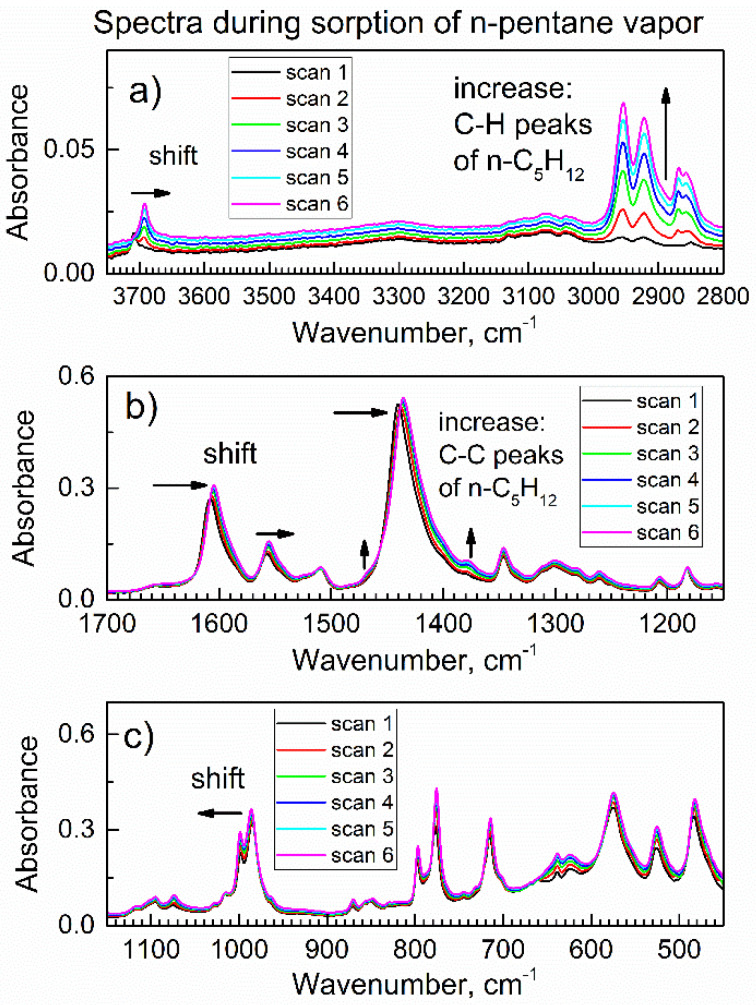
The *in-situ* time-dependent ATR-FTIR spectra of compound **2** in the flow of n-pentane vapor in dried air (time range 0-9.6 min.). (**a**) The high wavenumbers IR range; (**b**) the mid-IR range; (**c**) the low wavenumbers IR range.

Next, the flow of gas through the chamber was switched to purge gas (dried air saturated with n-pentane vapor). With the continuing flow of purge gas, *in-situ* ATR-FTIR spectra were continuously collected, see Figure 3; each spectrum took 1.6 min. Upon the exposure of compound **2** to the vapor of n-pentane in dried air, there were gradual changes in the spectra. First, in Figure 3a, the peak at 3708 cm^−1^ due to the stretch vibration of the free O–H group in compound **2** underwent a significant red shift (shown by horizontal arrow) to 3693 cm^−1^. At the same time, there was no increase in the characteristic peaks of adsorbed water molecules in the range 3600–3200 cm^−1^.

Additionally, in Figure 3b, there was no growth in the characteristic peak [31] at ca. 1650 cm^−1^ due to the deformation vibration of water molecules. This means that the observed shift in Figure 3a (shown by horizontal arrow) was due to the interaction of the O-H group in compound **2** with n-pentane upon its sorption. Second, in Figure 3a there was a significant and progressive growth of new peaks (shown by vertical arrow) within 3000–2800 cm^−1^, namely at 2954, 2922, 2869, and 2855 cm^−1^.

Figure S4 shows the ATR-FTIR spectrum of liquid n-pentane, where the spectral ranges were the same as in Figure 3. In Figure S4a, four peaks within the 3000–2800 cm^−1^ range belong to asymmetric and symmetric C-H vibrations of CH_3_ and CH_2_ groups in the n-pentane molecule [34]. Therefore, new peaks in Figure 3a at 2954, 2922, 2869, and 2855 cm^−1^ belong to the molecules of n-pentane absorbed by compound **2**. Additionally, one can see in Figure S4b the peaks at 1460 and 1379 cm^−1^, which correspond to twist and deformation vibrations of the CH_3_ group [34]. They can also be seen in the spectra of compound **2** with adsorbed n-pentane in Figure 3b as weak but growing shoulders (shown by vertical arrows).

During the collection of the subsequent six *in-situ* ATR-FTIR spectra of compound **2** in the flow of n-pentane (time range 9.7–19.2 min.), there was still some growth of peaks at 2954, 2922, 2869, and 2855 cm^−1^ (data not shown), but spectral changes stopped by the end of that time interval. This indicates the completion of sorption of n-pentane vapor by compound **2** within less than 20 min. During the subsequent purge of the flow chamber with the vapor of n-pentane in dried air, the spectra did not change.

**Figure 4:**
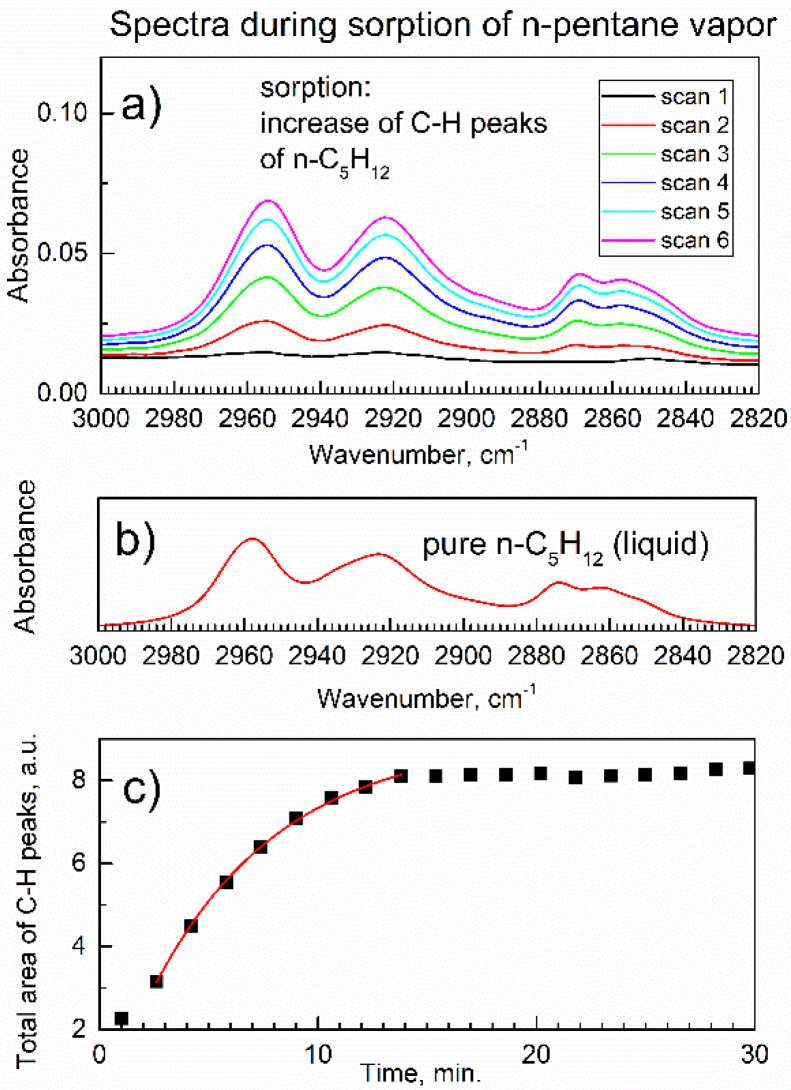
The time evolution of *in-situ* ATR-FTIR peaks for compound **2** in the flow of n-pentane vapor in dried air. (**a**) The C-H peaks of adsorbed n-pentane in the first 9.6 min. (**b**) The ATR-FTIR spectrum of pure n-pentane. (**c**) The integrated area of the C-H peaks of adsorbed n-pentane for each spectrum and numeric curve fitting (red line).

Figure 4a shows the ATR-FTIR spectra of C-H peaks due to n-pentane adsorbed by compound **2** during the first six scans (sorption time 0–9.6 min). Figure 4b shows, for comparison, the ATR-FTIR spectrum of pure n-pentane. Figure 4c shows areas of these C-H peaks of adsorbed n-pentane integrated within 3000–2820 cm^−1^ for each spectrum within 0–30 min. The total integrated area of the C-H peaks reflects the content of n-pentane adsorbate, and the time dependence in Figure 4c reflects the chemical kinetics of sorption.

Within the first ca. 15 min., there was a progressive increase in the integrated peak area, and then a plateau was achieved. This means achieving a dynamic equilibrium between compound **2** (sorbent) and the vapor of n-pentane (adsorbate) in the flow of dried air, Equation (1):

[Al-MOF-TCPPH_2_] (s) + x n-C_5_H_12_ (vap) → [Al-MOF-TCPPH_2_](n-C_5_H_12_)_x_ (s) (1)

The product in Equation (1) is an adsorption complex of compound **2** with n-pentane as “guest” molecules, but this adsorption complex was prepared under dynamic conditions, and its formal stoichiometric index x depends on the reaction progress.

Figure 4c also shows the numeric curve fitting (red line) of the early stage of sorption per Equation (1), using the formula of chemical kinetics of the pseudo-first-order rate law [35] where the amount of the product is y(t) = A + B × (1 − exp(−k × t)). In this model, the integrated area of C-H peaks of n-pentane in the adsorption complex is proportional to the molar amount of n-pentane in this complex which is, in turn, proportional to the molar amount of this complex. In the kinetic formula, k is the effective rate constant, B is the proportionality coefficient, and coefficient A is utilized to offset the initial mass of the sorbent. Since MOF is in the form of powder, the rate constant is likely affected by diffusion. This kinetic model was successfully utilized [9] in the kinetic study of the sorption of water vapor by super-hydrophilic MOF MIL-160(Al).

In Figure 4c, the integrated area of C-H peaks in the very first spectrum (adsorption time 0–1.5 min.) was omitted from the fitting. The kinetic curve in Figure 4c is very well modeled by the pseudo-first-order rate law with the adjusted goodness-of-fit parameter R^2^_adj_ = 0.99903. In the kinetic model described here, the amount of n-pentane in the adsorption complex was assumed to be linearly proportional to the integrated area of its C-H peaks in the ATR-FTIR spectrum of the complex. This assumption was verified by the direct method, *in-situ* time-dependent gravimetry under similar conditions, Section 3.4.

After about 35 min., the *in-situ* sorption experiment was stopped since all liquid n-pentane in a gas flush can (see Materials and methods) had evaporated. From this time, a purge gas flowing through the flow chamber with the specimen was dried air. The intensity of the characteristic C-H peaks of n-pentane within 3000–2800 cm^−1^ in the spectra of the adsorption complex slightly decreased but then remained constant (data not shown) for 50 scans (80 min). This indicates that the reverse process, namely the *in-situ* desorption of n-pentane vapor did not readily proceed when the surrounding flowing air had zero partial pressure of n-pentane. This indicates the relatively strong bonding of n-pentane to functional groups in the reactant (Al-MOF-TCPPH_2_).

### 3.3. The In-Situ Time-Dependent ATR-FTIR Spectra to Find Specific Functional Groups in Compound ***2*** Interacting with n-Pentane

**Figure 5:**
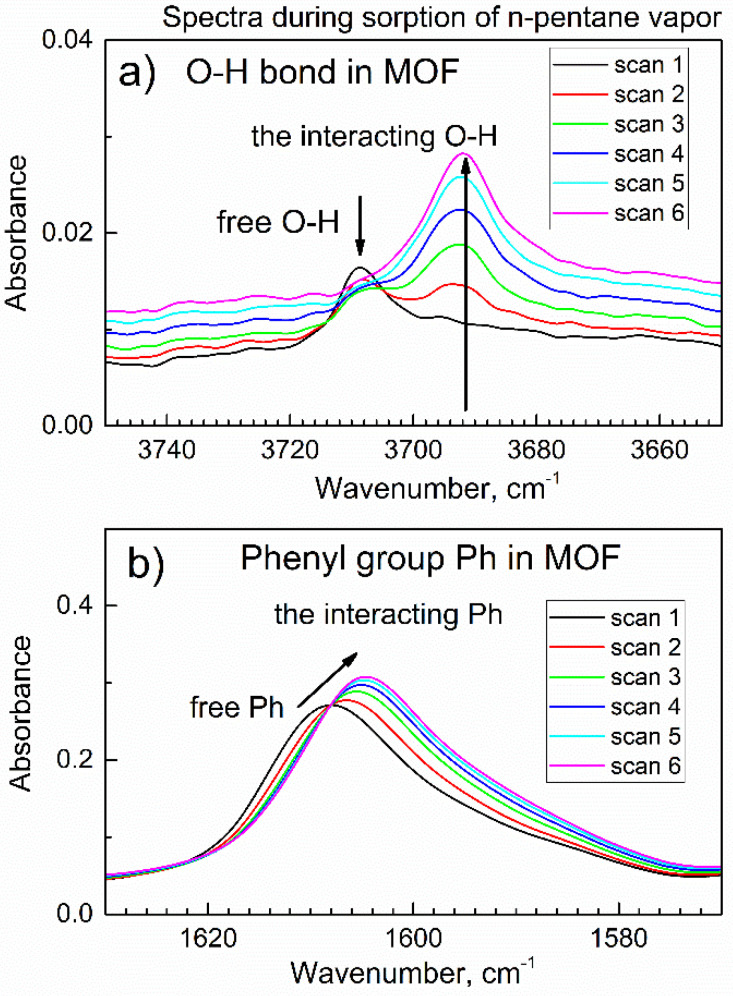
The time evolution of *in-situ* ATR-FTIR spectra of compound **2** in the flow of n-pentane vapor in dried air in the first 9.6 min. (**a**) Peak of the O-H group; (**b**) peak of the phenyl group.

Figure 5 shows the time evolution of selected ATR-FTIR spectral ranges of compound **2** during the gradual sorption of n-pentane vapor. Based on Equation (1), the product of sorption is the adsorption complex with variable content of n-pentane “guest” molecules.

Figure 5b shows that the peak of compound **2** at 1608 cm^−1^ due to the phenyl group is gradually shifting to 1604 cm^−1^ upon sorption (shown by arrow). This indicates that the phenyl group in compound **2** interacts with the n-pentane molecule. This finding is consistent with multiple reports of van der Waals interactions between molecules of aliphatic and aromatic hydrocarbons. For example, Menapace et al. [36] reported Van der Waals interactions in benzene-methane clusters.

In Figure 5a, the peak due to the stretching vibration of the free (non-bonded) O-H group in compound **2** was progressively and significantly shifted during the sorption of n-pentane, from 3708 cm^−1^ to 3692 cm^−1^. It is seen as the decrease of one respective peak and the increase of the other one (shown by vertical arrows). This indicates that the polar O-H group of compound **2** (the “host” material) interacts with chemical bonds in the non-polar n-pentane “guest” molecule.

**Figure 6:**
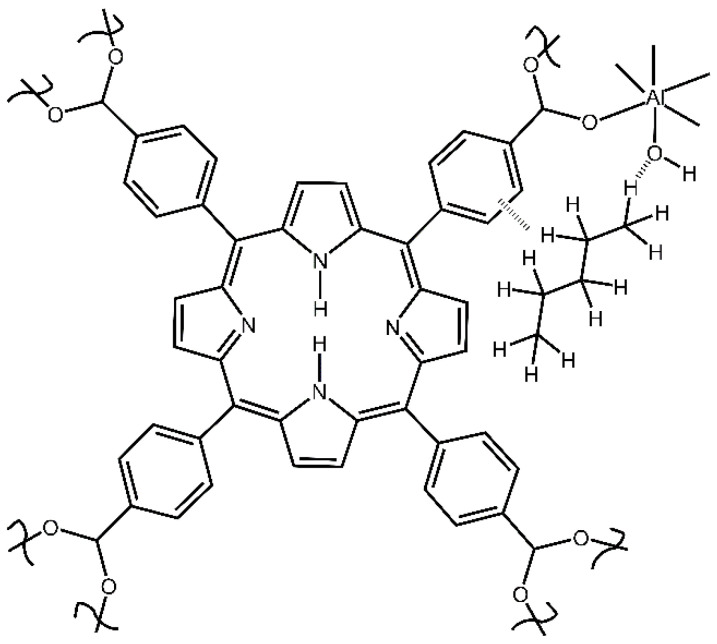
Bonding of n-pentane to the O-H and the phenyl group in compound **2**.

A suitable model was proposed by Huang et al. [37], who reported that several aluminum MOFs preferentially adsorb methane CH_4_ over nitrogen N_2_. The aluminum MOFs in ref. [37] contains, similarly to compound **2** in this work, the μ-OH group, and “The mechanism underlying the disparity of Al-MOFs affinity toward CH_4_ was deciphered via theoretical simulation, suggesting that the synergetic effects of accessibility of strong affinity sites (μ-OH) on AlO_6_ chains and polar pore surface induced by varying linkers highly promoted the CH_4_ uptake” [37]. Another relevant model was provided by Wischert et al. [38], who proposed a mechanism of interaction between the hydrated surface of aluminum oxide (alumina) Al_2_O_3_ and methane. Namely, water pre-adsorbed on alumina leads to surface hydroxyl groups Al-O-H which are structurally similar to the μ-OH groups in aluminum MOFs, including compound **2**. The “adsorbed water increases the reactivity of non-adjacent (Al,O) pairs towards C-H bond activation” [38]. Data in Figure 5 indicate that, in the adsorption complex, the n-pentane molecule interacts with the O-H group and phenyl group of the TCPPH_2_ linker; in Figure 6, only one Al site is shown for simplicity.

This interaction is apparently favored by the relatively large size of the n-pentane molecule with length estimates [16] of 6.9 Å and 7.7 Å. Additionally, the n-pentane molecule is able to be located within the large nanopore [39] of compound **2**, which has space group Cmmm and lattice parameters a = 31.978(3) Å, b = 6.5812(4) Å, c = 16.862(2) Å. It is also possible that the two sorption sites interact with n-pentane molecules.

It is important to note that the shift of the infrared peak due to the O-H group in compound **2** upon n-pentane sorption (Figure 5a) was not due to the sorption of a trace amount of water vapor in the dried air (carrier gas of n-pentane). First, we previously reported that the activated MOF compound **2** does not readily sorb water vapor, even in the relatively moist ambient air, as determined by *in-situ* time-dependent ATR-FTIR spectroscopy [31]. Second, in the independent experiment, a drop of liquid n-pentane was added to compound **2** on the ATR crystal, and a similar spectral shift of the peak due to the O-H group was immediately observed (data not shown). These findings indicate that the O-H group in the activated MOF compound **2** is involved in an interaction with n-pentane molecules. The somewhat surprising interaction of the n-pentane adsorbate with the polar O-H group in compound **2** needed additional investigation; this is described in Section 3.5.

**Figure 7:**
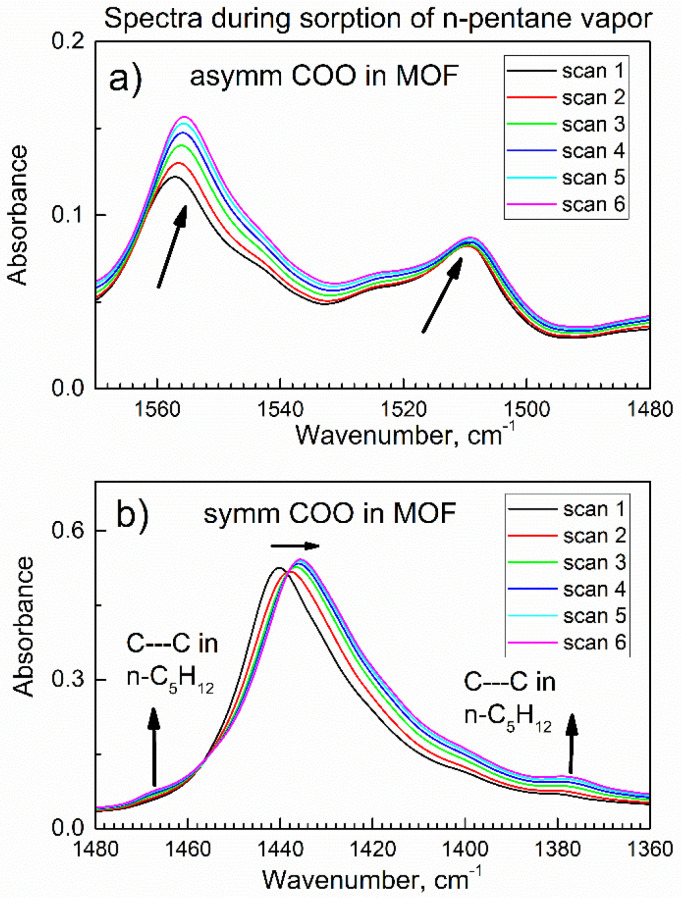
The time evolution of *in-situ* ATR-FTIR spectra of compound **2** in the flow of n-pentane vapor in dried air in the first 9.6 min. (**a**) peak of the asymmetric COO^−^ vibration; (**b**) peak of the symmetric COO^−^ vibration.

Figure 7 shows the evolution of *in-situ* ATR-FTIR spectral peaks due to vibrations of the carboxylate -COO^−^ group of compound **2** during the gradual sorption of n-pentane vapor. In Figure 7a, peaks due to the asymmetric vibrations of the -COO^−^ group are shifted to lower wavenumbers (shown by arrows), which is consistent with the bonding “guest” n-pentane molecule to these sorption sites. Figure 7b shows a shift (shown by horizontal arrow) of the peak due to the symmetric vibration of the -COO^−^ group when the sorption of n-pentane progressed. Interestingly, Figure 7b also shows weak but consistently increasing shoulders at 1460 and 1379 cm^−1^ (shown by vertical arrows) due to the twist and deformation vibrations of the -CH_3_ groups in n-pentane adsorbate [34].

**Figure 8:**
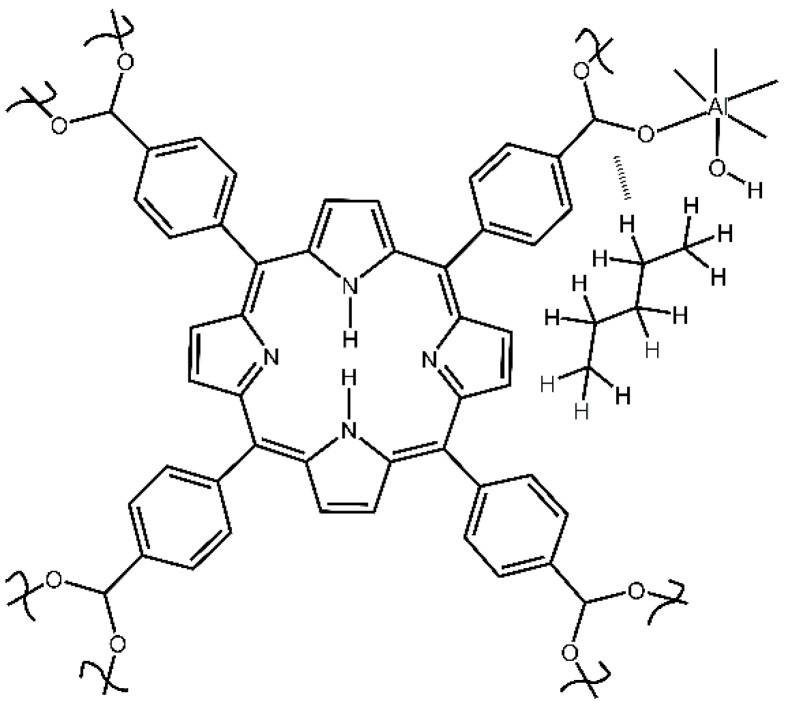
Bonding n-pentane to the -COO^−^ group in compound **2**.

These findings indicate that the n-pentane adsorbate “guest” molecule interacts with the carboxylate group, Figure 8. Bonding n-pentane to the -COO^−^ group in the MOF compound **2** is consistent with its bonding via the O-H group (Figure 6) since both groups are close to each other in the structure of this MOF; only one Al site is shown for simplicity.

### 3.4. Kinetics of Sorption of n-Pentane Vapor by Compound ***2*** Using In-Situ Time-Dependent Gravimetric Analysis

**Figure 9:**
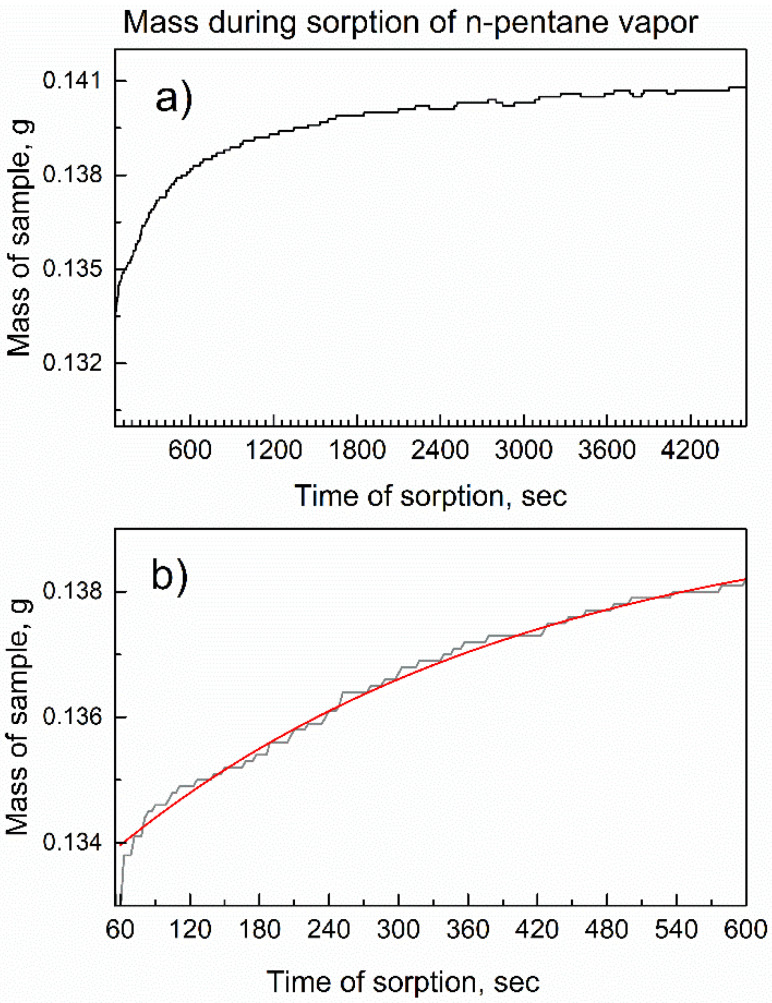
The *in-situ* time-dependent gravimetry of compound **2** in the flow of n-pentane vapor in dried air. (**a**) Dynamics of mass increase due to n-pentane sorption; (**b**) Sorption in the first 600 s and its kinetic analysis; red line is the numeric curve fit.

To our knowledge, no reports are available on the sorption or desorption of hydrocarbons on MOFs studied by *in-situ* time-dependent gravimetric analysis. Figure 9 shows the mass of compound **2** on a plate of analytical balance (with its doors closed) as a function of time when the flow of vapor of n-pentane in the dried air was passed over the sample.

The mass gradually increased and then reached a plateau, as shown in Figure 9a, achieving the dynamic equilibrium of sorption in Equation (1). The determination of the chemical composition of the adsorption complex was conducted as follows. The reactant was compound **2**, which contain [TCPPH_2_]^4−^ anions (formed by the dissociation of four carboxylic groups of precursors of the linker), as shown in Figure 1. For clarity, H_2_ in [TCPPH_2_]^4−^ denotes the remaining non-dissociated protons connected to nitrogen atoms in the porphyrin ring. In the structure of compound **2**, tetra-anions [TCPPH_2_]^4−^ are connected to the Al-O-H units, thus giving the simplified formula [TCPPH_2_^4−^]_1_(Al^3+^)_2_(OH^−^)_2_. This corresponds to the Hill formula C_48_H_28_N_4_O_10_Al_2_ and formal molar mass 874.7 mg/mmol. Upon dynamic sorption, the reactant (compound **2**) of mass 0.1329 g (0.152 mmol) absorbed 0.0078 g n-pentane (0.108 mmol). This corresponds to Equation (2):

0.152 [Al-MOF-TCPPH_2_] (s) + 0.108 n-C_5_H_12_ (v) → [Al-MOF-TCPPH_2_]_0.152_(n-C_5_H_12_)_0.108_ (s) (2)

The re-calculation of the stoichiometry gives the formula [Al-MOF-TCPPH_2_]_1_(n-C_5_H_12_)_0.71_. The non-integer in the formula likely indicates that more n-pentane can be adsorbed; this hypothesis was tested in Section 3.5.

Figure 9b shows the kinetic curve of an early stage of sorption and its numeric curve fitting, using a formula [35] for the pseudo-first-order rate law by the product y(t) = A + B × (1 − exp(−k × t)). In the formula, k is an effective rate constant, B is the proportionality coefficient, and A coefficient accounts for the initial amount of a reactant. The dependence in Figure 9b was fitted well by the pseudo-first-order rate law, with the adjusted goodness-of-fit parameter R^2^_adj_ = 0.99485 and kinetic rate constant of sorption k(s) = 3.01 × 10^−3^ ± 4.55 × 10^−5^ min^−1^. It is important to note that fluctuations of mass in Figure 9 are very minor, with an increment of 0.1 mg, consistently and with the accuracy of analytical balance. This indicates that the kinetics of sorption could be determined with high accuracy and consistently with a very good R^2^_adj_ parameter.

### 3.5. The Ex-Situ (Static) Sorption of n-Pentane Vapor in Dried Air by Compound ***2***

After the static sorption of n-pentane vapor in the desiccator (see Materials and methods), the obtained sample was removed from the desiccator and promptly weighed. The mass had increased vs. that of compound **2**, indicating the sorption of n-pentane. Sorption under the static conditions described here occurs under equilibrium, and the adsorbed amount of n-pentane is different from that obtained using the analytical balance as the flow chamber, Section 3.4. Upon static sorption, the specimen of compound **2** with mass 0.0783 g (0.0895 mmol) adsorbed 0.0228 g of n-pentane (0.3160 mmol):

0.0895 [Al-MOF-TCPPH_2_] (s) + 0.3160 n-C_5_H_12_ (v) → [Al-MOF-TCPPH_2_]_0.0895_(n-C_5_H_12_)_0.3160_ (s) (3)

The re-calculation of the stoichiometry of the obtained product compound **3** gives formula [Al-MOF-TCPPH_2_]_1_(n-C_5_H_12_)_3.5_ or [Al-MOF-TCPPH_2_]_2_(n-C_5_H_12_)_7._ The stoichiometric index 7 in the formula was likely due to the sharing of the n-pentane adsorbate molecule by two neighboring units of compound **3**. A similar geometry [16] was reported for n-pentane adsorbed in the nanopores of certain MOFs. Compound **3**, prepared during static sorption, was unstable in ambient air, gradually losing n-pentane to ambient air; the kinetics of this loss is analyzed in Section 3.6.

**Figure 10:**
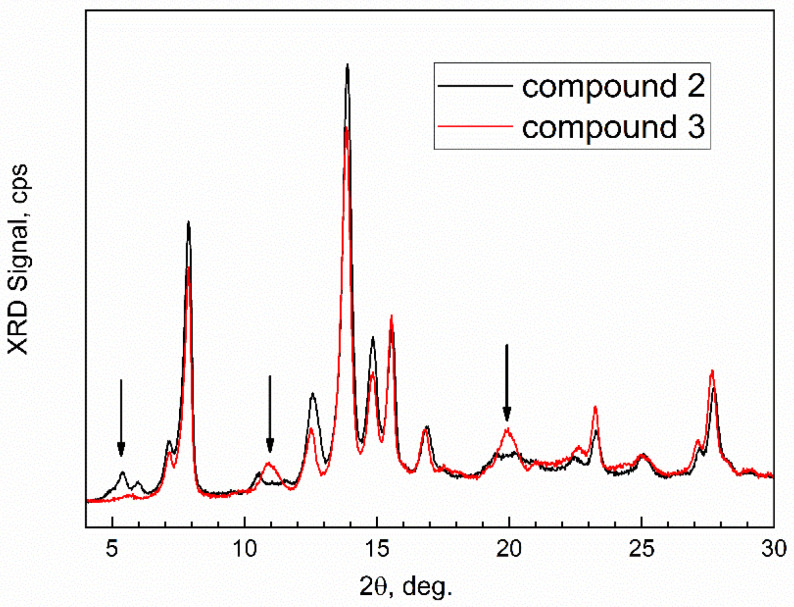
Powder XRD patterns of compound **2** and compound **3**.

The freshly prepared compound **3** on the XRD plate was promptly transferred to a specimen compartment of the XRD instrument. Its XRD pattern is shown in comparison with that of the activated MOF compound **2**, Figure 10.

The patterns show distinct differences at ca. 2θ = 5 deg., 11 deg. and 20 deg. which indicates the formation of a new compound with a slightly distorted crystalline lattice compared to compound **2**. This can be interpreted as the “breathing” of the framework of compound **2** upon the inclusion of n-pentane “guest” molecules to nanopores. Due to the instability of compound **3** during the XRD experiment, the modeling of its lattice was not performed. On the other hand, compound **2** has a lattice of the orthorhombic space group Cmmm and large [39] lattice parameters a = 31.978(3) Å, b = 6.5812(4) Å, c = 16.862(2) Å. This Al-MOF could be classified as mesoporous MOF, and such a large nanopore (estimated volume of about 3500 cubic Å) can accommodate more than two n-pentane molecules of ca. 6.8 Å in length.

The presented data show that compound **2** is promising for applications based on the sorption of vapor of pentane and other light hydrocarbons. On one hand, it shows the significant adsorbed amount; 0.0783 g sorbent compound **2** adsorbs 0.0228 g n-pentane “guest”, giving a high adsorbed amount of 29.1 wt.%. On the other hand, sorption is easily reversible by switching the flow of gas, and both the sorption and desorption of n-pentane can facilely occur at an ambient temperature. Hence, compound **2** has potential in small-scale industrial applications, such as the purification and separation of light hydrocarbons and chemo-sensing.

### 3.6. Kinetics of Desorption of n-Pentane by Compound ***3*** by In-Situ Time-Dependent Gravimetric Analysis

**Figure 11:**
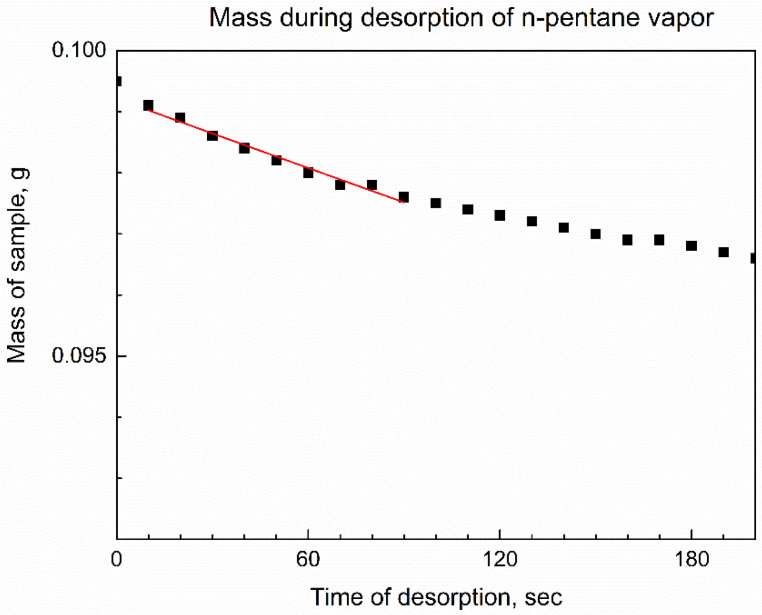
The *in-situ* time-dependent gravimetry measurement of compound **3** in the flow of dried air during n-pentane desorption, and its kinetic analysis (red line is the numeric curve fit).

Figure 11 shows the mass of compound **3** on a plate of analytical balance vs. time when the interior of the balance, including the surroundings of the specimen, was purged with dried air. The mass is shown to be decreasing, and the numeric fitting of the initial data points was performed by the formula of the pseudo-first-order rate law [35] by the reactant, namely y(t) = A × exp(−k × t). Here, k is an effective rate constant, and A is the initial amount of reactant. The fitting has an adjusted goodness-of-fit parameter R^2^_adj_ = 0.97348 and the obtained desorption rate constant is k(des) = 1.92 × 10^−4^ ± 1.12 × 10^−5^ min^−1^.

The MOF in this work contains porphyrin linker and polar H-O-(Al) sites and shows the capability to sorb the vapor of the hydrophobic non-polar compound n-pentane; the sorption of the vapor of the polar hydrophilic compound water was shown earlier [31]. Studies of competitive sorption are in progress. Further, this work revealed advanced and previously unexplored capabilities of a novel method of *in-situ* time-dependent ATR-FTIR spectroscopy when it is coupled with studies of the sorption and desorption of vapor in a controlled atmosphere. The mechanistic details of sorption, such as specific binding sites in the MOF sorbent with the porphyrin linker of a complex structure and sorption kinetics, were revealed. Further, the kinetics of sorption and desorption was determined by the complementary *in-situ* time-dependent gravimetry which was also conducted in a controlled atmosphere. The described powerful dual experimental approach is facile and it shows high promise in the mechanistic studies of sorption and desorption by other MOFs and various functional solids, when they interact with molecules in the gas (vapor) phase.

## 4. Conclusions

The methodology of *in-situ* time-dependent ATR-FTIR spectroscopy of sorption in a controlled atmosphere was described. It was successfully tested in the mechanistic study of the sorption and desorption of n-pentane vapor on porphyrin Al-MOF compound **2** in the simple gas flow chamber attachment. The *in-situ* time-dependent ATR-FTIR spectra demonstrated how compound **2** readily sorbed the vapor of n-pentane in the flowing dried air under dynamic sorption conditions, following the pseudo-first-order rate law and forming an adsorption complex. In it, n-pentane adsorbate molecules were are weakly bonded to the phenyl group and carboxylate anion of the linker, and the O-H group is involved. Chemical kinetics of the sorption, per the complementary *in-situ* time-dependent gravimetry, proceeds by the pseudo-first-order rate law with a rate constant of sorption k(s) = 3.01 × 10^−3^ min^−1^. Upon static sorption (no gas flow) under equilibrium conditions, compound **2** sorbs n-pentane vapor in dried air, forming an adsorption complex (compound **3**) with the formula [Al-MOF-TCPPH_2_]_2_(n-C_5_H_12_)_7._ The XRD data suggest the “breathing” of the framework of this MOF to accommodate n-pentane “guest” molecules. Further, when compound **3** was exposed to the flow of dried air, it facilely lost part of adsorbed n-pentane, and the kinetics of desorption followed the pseudo-first-order rate law and had a rate constant k(des) = 1.92 × 10^−4^ min^−1^. The significant and specific advantage of the described method of *in-situ* time-dependent ATR-FTIR spectroscopy in a controlled atmosphere is the capability to facilely study the mechanism and kinetics of the sorption and absorption of target molecules in the vapor phase, while eliminating the interfering effects of humidity and other common environmental factors. Further, the combination of *in-situ* time-dependent ATR-FTIR spectroscopy and gravimetry, both conducted in a controlled atmosphere, is a new powerful experimental approach with significant capabilities for research and engineering. Additionally, the elimination of environmental humidity allows facile and reliable studies of adsorbates that form weak bonds with the “host” lattice of the sorbent, when humidity is usually a problem due to the competing sorption of water. A controlled atmosphere in a limited reactor space allows facile and safe studies of the sorption and desorption of hazardous chemicals in the form of gas or vapor. The described facile yet highly capable methodology could be utilized in virtually every lab to study the chemical mechanisms of processes of sorption and desorption, the stoichiometry of complexes of porous materials with “guest” molecules, and the kinetics of “solid–gas” interactions.

## Data Availability

The data presented in this study are available in this article and supplementary material.

## Supplementary Materials

The following supporting information can be downloaded at: https://www.mdpi.com/article/10.3390/nano13091529/s1, Figure S1. Generic schematics of typical ATR accessory. Figure S2. The simplified drawing of the flow chamber. Figure S3. Relative humidity (RH) inside the flow chamber during pre-purge with dried air. Figure S4. (a–c) The ATR-FTIR spectrum of liquid n-pentane.
