# Supplementary material for: Sorption and Desorption of Vapor of n-Pentane by Porphyrin Aluminum Metal–Organic Framework: Mechanism of Bonding, Kinetics and Stoichiometry by Complementary In-Situ Time-Dependent and Ex-Situ Methods
Source: Nanomaterials (Basel). 2023 May 1;13(9):1529. doi: 10.3390/nano13091529 (PMC10180467; doi:10.3390/nano13091529)
Supplement: Supplementary file 1 [file nanomaterials-13-01529-s001.zip › nanomaterials-2316124-supplementary.pdf]

nanomaterials

Supplementary Materials

Sorption and Desorption of Vapor of n-Pentane by Porphyrin Aluminum Metal–Organic Framework: Mechanism of Bonding, Kinetics and Stoichiometry by Complementary *In-Situ* Time-Dependent and *Ex-Situ* Methods

Georgia-Annicette Banga-Bothy and Alexander Samokhvalov *

Department of Chemistry, Morgan State University, 1700 East Cold Spring Lane, Baltimore, MD 21251, USA
* Correspondence: alexandr.samokhvalov@morgan.edu

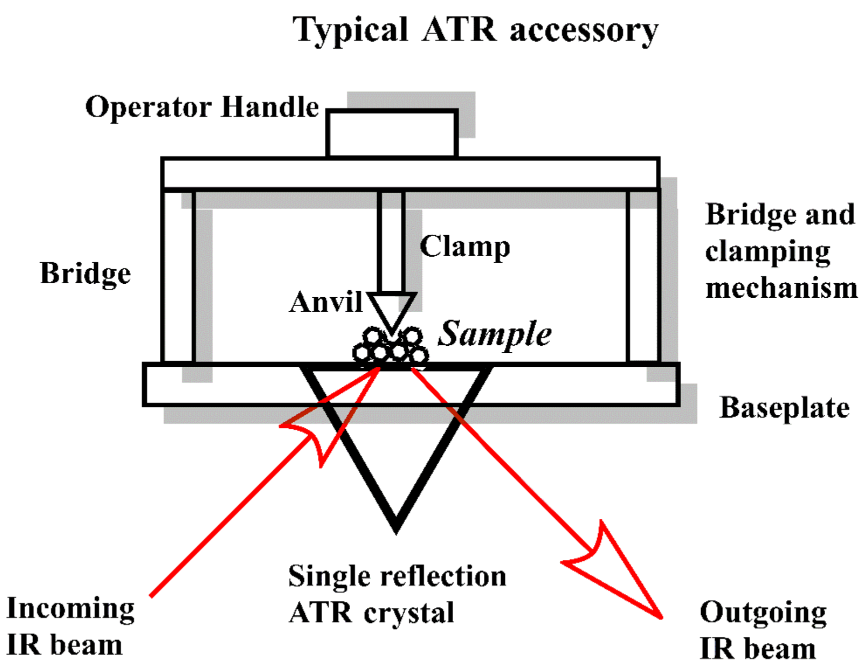

Figure S1. Generic schematics of typical ATR accessory.

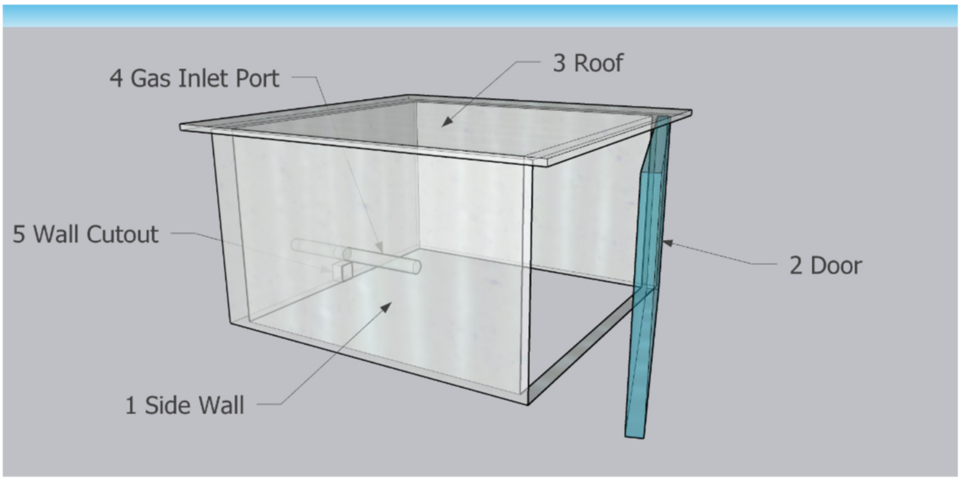

Figure S2. The simplified drawing of the flow chamber.

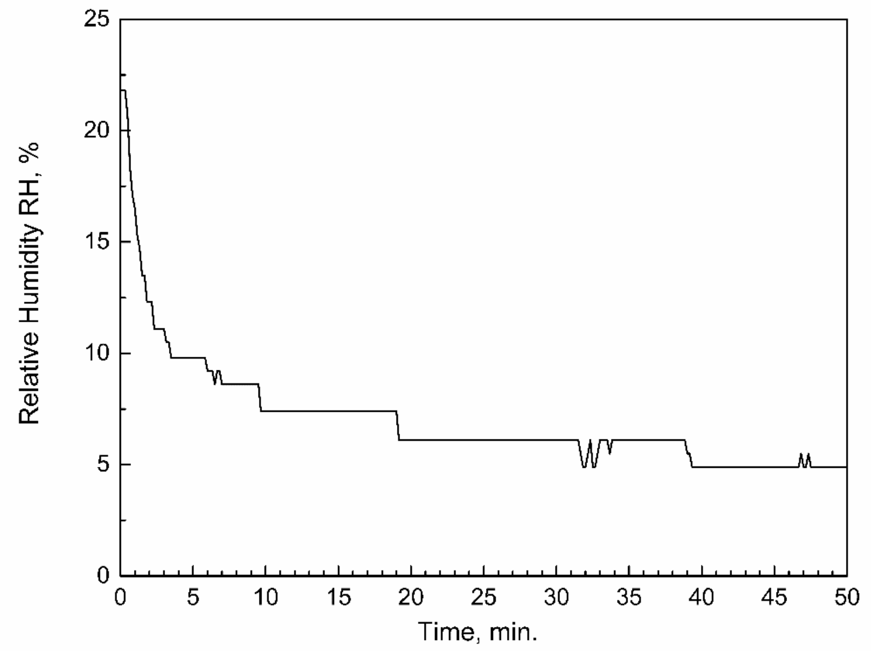

Figure S3. Relative humidity (RH) inside the flow chamber during pre-purge with dried air.

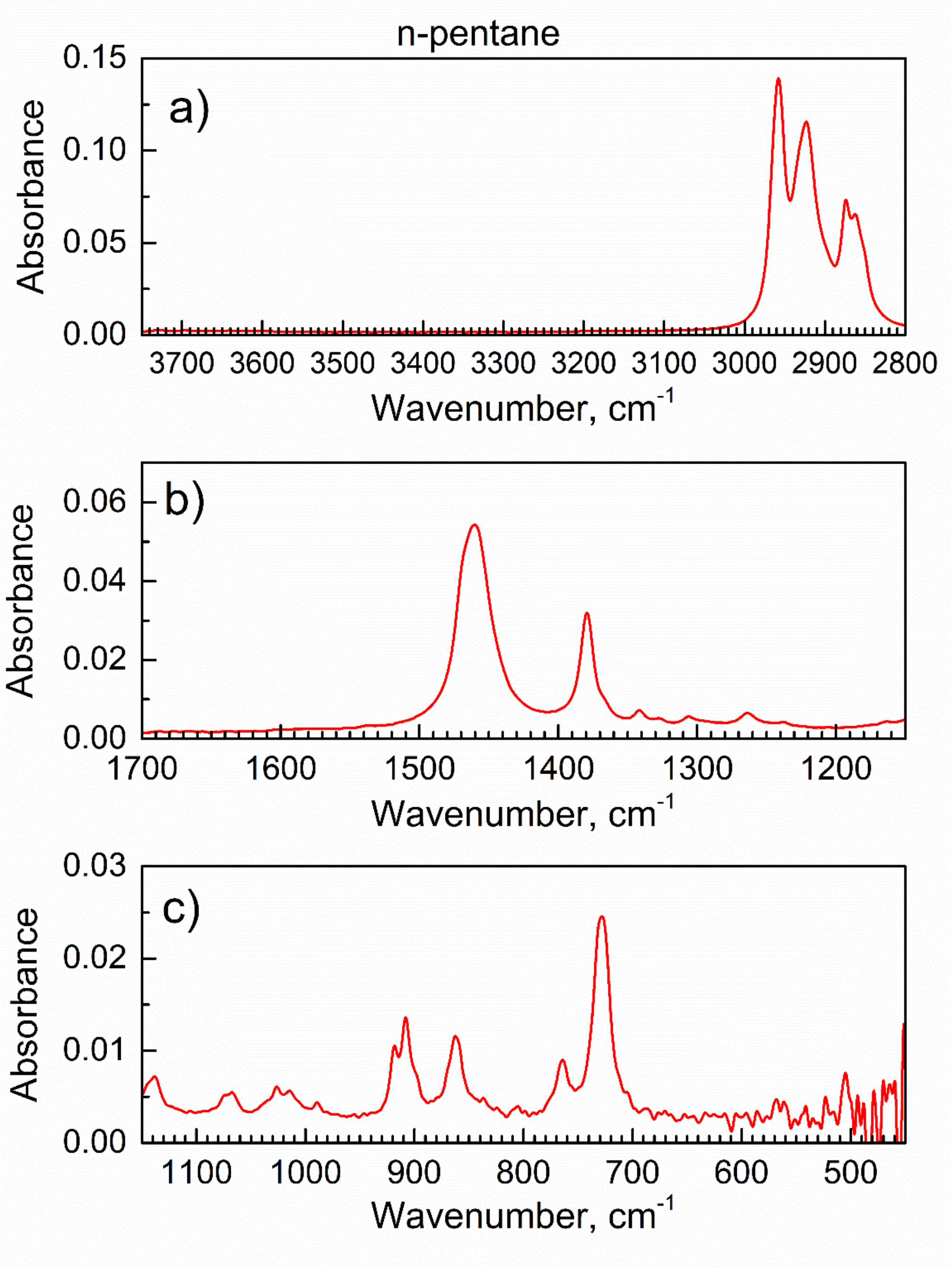

Figure S4. The ATR-FTIR spectrum of liquid n-pentane. (**a**) The high wavenumbers IR range; (**b**) the mid-IR range; (**c**) the low wavenumbers IR range.
